# Flow perfusion rate modulates cell deposition onto scaffold substrate during cell seeding

**DOI:** 10.1007/s10237-017-0985-4

**Published:** 2017-11-29

**Authors:** A. Campos Marín, M. Brunelli, D. Lacroix

**Affiliations:** Department of Mechanical Engineering, Insigneo Institute for in Silico Medicine, The University of Sheffield, Pam Liversidge Building, Mappin Street, Sheffield, S1 3JD UK

**Keywords:** Scaffold, Cell seeding, Computational fluid dynamics, Particle modelling, Microfluidics, Perfusion bioreactor

## Abstract

The combination of perfusion bioreactors with porous scaffolds is beneficial for the transport of cells during cell seeding. Nonetheless, the fact that cells penetrate into the scaffold pores does not necessarily imply the interception of cells with scaffold substrate and cell attachment. An in vitro perfusion system was built to relate the selected flow rate with seeding efficiency. However, the in vitro model does not elucidate how the flow rate affects the transport and deposition of cells onto the scaffold. Thus, a computational model was developed mimicking in vitro conditions to identify the mechanisms that bring cells to the scaffold from suspension flow. Static and dynamic cell seeding configurations were investigated. In static seeding, cells sediment due to gravity until they encounter the first obstacle. In dynamic seeding, 12, 120 and 600 $\upmu \hbox {l/min}$ flow rates were explored under the presence or the absence of gravity. Gravity and secondary flow were found to be key factors for cell deposition. In vitro and in silico seeding efficiencies are in the same order of magnitude and follow the same trend with the effect of fluid flow; static seeding results in higher efficiency than dynamic perfusion although irregular spatial distribution of cells was found. In dynamic seeding, 120 $\upmu \hbox {l/min}$ provided the best seeding results. Nevertheless, the perfusion approach reports low efficiencies for the scaffold used in this study which leads to cell waste and low density of cells inside the scaffold. This study suggests gravity and secondary flow as the driving mechanisms for cell-scaffold deposition. In addition, the present in silico model can help to optimize hydrodynamic-based seeding strategies prior to experiments and enhance cell seeding efficiency.

## Introduction

Cell seeding of 3D scaffolds precedes the steps involved in the formation of living functional tissues. Bioreactors should provide a controlled environment which ensures adequate seeding outcomes for subsequent tissue development. The initial number of cells and spatial distribution inside the scaffold unit is strongly related to tissue growth and final tissue properties (Holy et al. 2000; Saini and Wick 2003). Furthermore, cell seeding efficiency should be maximized to avoid the waste of donor cells (Vunjak-Novakovic et al. 1998). Up to now, perfusion systems seem to be the preferred solution to meet those requirements due to their excellent mass transfer properties and the induced shear stress which is beneficial for cell activity (Wendt et al. 2003; Zhao et al. 2007; Choi et al. 2008; Sonnaert et al. 2014). Nonetheless, the enhancement of mass transfer can imply high shear stresses which can be detrimental for cell-scaffold adhesion (Lu et al. 2004). Various flow rates are usually investigated to achieve optimal seeding conditions (Du et al. 2009; Koch et al. 2010). However, performing those in vitro trials can involve high costs and time. In addition, visual access inside the scaffold is limited due to the opacity of most of the scaffold materials. In silico models can serve as a virtually unlimited source of trials and help to optimize seeding systems prior to in vitro experiments.

For instance, a computational cell seeding model developed by Olivares and Lacroix (2012) reported the optimal seeding time under specific seeding conditions in order to perform time effectively without losing resources. Moreover, they provided hints for scaffold design by reporting the effect of pore size on the final distribution of cells inside the scaffold. Adebiyi et al. (2011) used a computational model to find the pressure gradient value in a vacuum-induced suction system in order to achieve a homogenous cell distribution inside the scaffold. Nevertheless, these models are tested for specific scenarios and their versatility to explore other seeding conditions should be investigated. As an illustration, in the study of Adebiyi et al. (2011), the actual scaffold microstructure is not included in the model which would be essential to elucidate the effect of pore size and shape on cell seeding distribution (Olivares and Lacroix 2012). In addition, Olivares and Lacroix did not include the effect of gravity in cells trajectory which has a major role for lower flow rates as seen in the study of by Campos Marin et al. (2017) where cell tracking experiments were performed inside a 3D porous scaffold.

Herein, an in vitro seeding system is built to understand the effect of different flow rates on cell adhesion and cell seeding efficiency. However, the in vitro system only permits to relate the inlet flow rate with final cell seeding efficiency. The goal of this study is to develop a computational model to predict the position of cells over time and space within a real scaffold under bioreactor conditions. The computational model aims to simulate cell seeding mimicking the in vitro system in order to reveal how the flow rate modulates the transport and deposition of cells onto the scaffold substrate. This model represents cells as a discrete phase suspended in the fluid phase as shown by Olivares and Lacroix (2012) and Adebiyi et al. (2011) with the difference that it includes the effect of gravity on cell motion. The mechanisms that bring cells to the scaffold are investigated for static and dynamic seeding. This in silico tool aims to provide a better insight of the events that may occur inside the microfluidic system and scaffold during seeding and help to optimize seeding strategies before experimental trials.

## Methods

### In vitro cell seeding

**Fig. 1:**
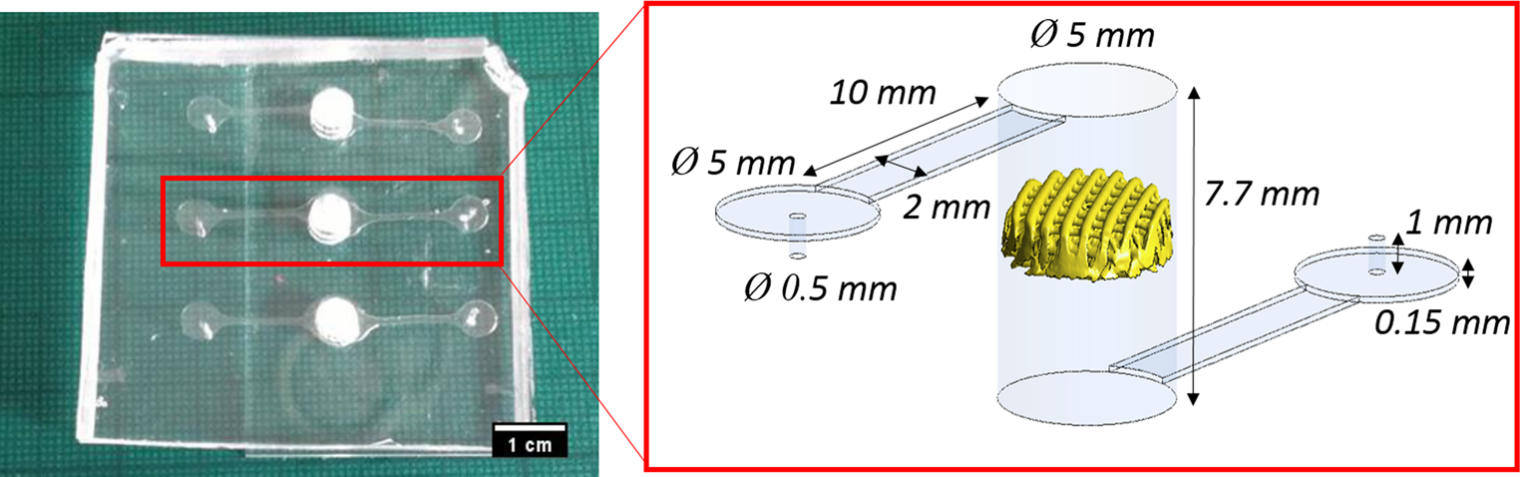
In vitro microfluidic seeding system with parallel configuration fitting three independent scaffolds in the same device (left). CAD-based representation of one chamber with a scaffold digitally reconstructed based on $\upmu \hbox {CT}$ images (right)

A microfluidic chamber made of polydimethylsiloxane (PDMS) was built with the dimensions indicated in Fig. 1. A commercial porous scaffold from 3D Biotek (New Jersey, USA) was selected for this study. The scaffold is made of polycaprolactone (PCL) and it has a regular porous microstructure formed by layers of cylindrical fibres with $300\,\upmu \hbox {m}$ diameter and with a distance of $300\,\upmu \hbox {m}$ between fibres. It has six layers with an offset of 90 degrees in the orientation of the fibres from layer-to-layer. In addition, there is a displacement of $300\,\upmu \hbox {m}$ among alternative layers. The scaffold has a cylindrical shape with 5 mm diameter and 1.5 mm height. The surface area and volume measured on 14 samples in a previous study are $153.85 \pm 17.39\,\hbox {mm}^{2}$ and $14.03\,\pm 1.79\,\hbox {mm}^{3}$ (Brunelli et al. 2017a).

Static and dynamic approaches were investigated for scaffold cell seeding. Cells were cultured until confluent in $\alpha$-mem culture media supplied with 10% FBS and 1% penicillin/streptomicine. For the static case, a $20\,\upmu \hbox {l}$ drop of human embryonic stem cells derived mesenchymal progenitor 002.5 (hMSCs) (Karlsson et al. 2009) suspended in medium with $10^{6}$ cells/ml concentration was located at the top of the scaffold. Then, the results were analysed after 1.5 h incubation under standard culture conditions to evaluate the effect of cell sedimentation due to gravity.

For the dynamic strategy, cell seeding by perfusion during 2 h was investigated for three different flow rates: 12, 120 and $600\,\upmu \hbox {l/min}$. These flow rates induced shear stresses up to 2 Pa in the scaffold which is optimal for cell attachment (Cherry 1993). Two syringe pumps were connected at the inlet and outlet to generate an alternate flow. Flow direction changed every $500\,\upmu \hbox {l}$ of dispensed fluid in order to maintain cells inside the microfluidic chamber and force them to pass through the scaffold pores several times.

Cell seeding efficiency was quantified by DNA assay (QuanIT DNA kit, Sigma Aldrich). Samples were cut into four pieces and placed in Eppendolf tubes. $200\,\upmu \hbox {l}$ of 0.5% trypsin was added following incubation for 5 min. Then, $200\,\upmu \hbox {l}$ of culture media was added to samples to block the action of trypsin and prevent damage of the cellular membrane. In order to achieve complete detachment of cells, samples underwent 5 s vortex. After 5 min, the solution was thoroughly mixed by pipetting several time. Then, $20\,\upmu \hbox {l}$ of suspension were tested for DNA quantification adding $180\,\upmu \hbox {l}$ of working solution made of lysis buffer and PicoGreen fluorescent stain (200:1 v/v). The same procedure was performed on wells where samples were stored to account for any cell left in the media or attached to the well rather than the scaffold. After 10 min in a dark environment, fluorescence was read by microplate reader at ex/em 485/535 nm.

### In silico cell seeding

#### Scaffold and chamber design

The boundary conditions of the in vitro microfluidic system were recreated for the computational model using the Design modeller workpackage from Ansys (Pennsylvania, USA). One scaffold sample was scanned with $\upmu \hbox {CT}$ and then digitally reconstructed using Simpleware (Synopsys, USA) following the same procedure described in Campos Marin and Lacroix (2015) (see Fig. 1).

#### Fluid domain

The CAD-based microfluidic chamber and the $\upmu \hbox {CT}$-reconstructed scaffold were imported into Icem Ansys to mesh the fluid volume with tetrahedral elements. After a mesh sensitivity analysis, the fluid domain was meshed applying a maximum length for the side of the tetrahedral elements of 50 and $20\,\upmu \hbox {m}$ for the elements outside and inside the scaffold, respectively. The mesh was generated with around 15 million tetrahedral elements using the ICEM robust octree algorithm which ensures a smooth transition from the elements far from the scaffold to the elements close to it.

#### Numerical simulations

The culture medium was modelled with water properties (viscosity of 0.001 $\hbox {Pa}\,\hbox {s}$ and a density of 1000 $\hbox {kg/m}^{3})$. The fluid flow was described by the continuity and 3D Navier Stokes equations. No-slip wall condition was considered at the channel boundaries and on the scaffold surface. In the static cell seeding simulation, a steady static case with zero velocity at the inlet and outlet boundaries was established. For the dynamic seeding, 12, 120 and $600\,\upmu \hbox {l}$/min flow rates were applied which correspond with of 0.1, 1 and 5 mm/s fluid velocity at the scaffold entrance, respectively. The fluid flow was imposed to change the direction of the flow every $500\,\upmu \hbox {l}$ of fluid dispensed. This is equivalent to change the direction of the flow every 2500, 250 and 50 s for the flow rates 12, 120 and $600\,\upmu \hbox {l}$/min, respectively.

Cells suspended in the culture medium were described as a discrete phase of sphere particles with $10\,\upmu \hbox {m}$ of diameter. Cell density was assumed to be higher than the density of the fluid phase. The density value selected was 1130 $\hbox {kg/m}^{3}$ as suggested in the literature (Bryan et al. 2014; Zhao et al. 2014). The discrete phase model from Fluent Ansys 15.0 was used to simulate cell trajectory in the fluid phase. The model tracks the particles along the previously calculated continuous phase by integrating the force balance on the particle, which is written in a Lagrangian frame. This force balance equates the particle inertia with the forces acting on the particle as described in Eq. 1.

1 $$\begin{aligned} \frac{\hbox {d}u_\mathrm{p}}{\hbox {d}t}=\frac{18\eta }{\rho _\mathrm{p} d_\mathrm{p}^{2}}\frac{C_\mathrm{d} Re}{24}(u-u_\mathrm{p}) + \frac{g(\rho _\mathrm{p}-\rho )}{\rho _\mathrm{p}} \end{aligned}$$

where $\eta$ is the fluid dynamic viscosity, $\rho$ is the fluid density, $\rho _\mathrm{p}$ is the density of the particle, $d_\mathrm{p}$ is the diameter of the particle, *u* is the local fluid velocity and $u_\mathrm{p}$ is the particle velocity. *Re* is the relative Reynolds number as result of the relative velocity of the cell phase with respect to the fluid phase and $C_\mathrm{d}$ is an empirical drag coefficient factor for spherical particles. The first term on the right represents the fluid drag force and the second term is the effect of gravity on cell trajectory due to the difference in densities between the discrete and the continuous phases. Cell seeding simulations were also carried out in the absence of gravity for the three flow rates investigated.

One-way coupling between phases was modelled where only the fluid phase had effect on the discrete phase due to the fact that the cell phase was too diluted representing only a 7.5% of total volume in the medium. Thus, cell-to-cell interactions were neglected considering that cells have sufficient time to respond to the local dynamic forces before any subsequent collision.

One cell was injected per surface mesh element in the microfluidic chamber with zero initial velocity. A total of 20,000 cells were injected in static seeding which corresponds to the number of cells found in vitro in a drop of $20\,\upmu \hbox {l}$ with assuming the cells were homogenously distributed in 10$^{6}$ cells/ml concentration. Cells were injected in the static fluid domain from the top of the central cylinder where the scaffold is placed. In dynamic seeding, the flow reverses every $500\,\upmu \hbox {l}$ of fluid dispensed which corresponds with 500,000 cells based on a homogeneous 10$^{6}$ cells/ml concentration. However, simulating 500,000 cells in silico was unaffordable with the computational resources available. Thus, 20,000 cells were simulated, same as for static seeding. A sensitivity test was performed to ensure that increasing the number of cells does not influence seeding efficiency results. A total of 72 injections were required over time to inject $\sim$ 20,000 cells at the inlet boundary which was defined with 274 surface mesh elements. The time step for the consecutive injections was 15, 1.25 and 0.35 s for the 12, 120 and $600\,\upmu \hbox {l}$/min flow rate cases, respectively. Two hours cell seeding were simulated as in the laboratory experiments. The interception of cells with the scaffold surface was quantified over time. It is noteworthy that cells were trapped in the scaffold surface as soon as they contacted it and removed from the computational calculations, for both static and dynamic seeding. Hence, no cell adhesion events or cell detachment were simulated. The same was applied in the chamber walls with the exception of the inlet and outlet boundaries where cells were subjected to reflection.

#### Cell trajectory in the absence of gravity

In the absence of gravity, cells follow the fluid streamlines for the three flow rates applied. This can be understood by the Stokes number (*Stk*) which can quantify the inertia of cells suspended in a fluid flow. It is a non-dimensional parameter that relates the characteristic time of cells $t_\mathrm{c}$ to the characteristic time associated with the flow field $t_\mathrm{f}$ (see Eq. 2). If $Stk<< 1$, cells follow the fluid streamlines and if *Stk* was $>>$ 1, inertia dominates cell motion as cells do not have time to respond to fluid velocity variations so they detach from the flow.

2 $$\begin{aligned} Stk =\frac{t_\mathrm{c}}{t_\mathrm{f}} \end{aligned}$$

$t_\mathrm{f}$ is described in Eq. 3 and it depends on the geometry of the obstacle $L_\mathrm{o}$ that the fluid flow encounters and the flow velocity far from the obstacle $U_\mathrm{o}$. In this case, $t_\mathrm{f}$ will vary throughout the seeding chamber. The case in which higher inertia in cell motion is expected is when $600\,\upmu \hbox {l}$/min flow rate is applied and cells enter in the cylindrical chamber where the scaffold is placed coming from the rectangular microchannel. Under those conditions, $U_\mathrm{o}$ is 50 mm/s and $L_\mathrm{o}$ is 5 mm and therefore; $t_\mathrm{f}$ is 0.1 s.

3 $$\begin{aligned} t_\mathrm{f}= \frac{L_\mathrm{o}}{U_\mathrm{o}} \end{aligned}$$

The characteristic time of cells which refers to the time required for them to respond to changes in the fluid flow is defined in Eq. 4:

4 $$\begin{aligned} t_\mathrm{c} = \frac{\rho _\mathrm{c} D^{2}}{18 \mu _\mathrm{f}} \end{aligned}$$

where $\rho _\mathrm{c}$ is the cell density, *D* is the cell diameter and $\mu _\mathrm{f}$ is the dynamic viscosity of the fluid. In this microfluidic seeding system, $t_\mathrm{c}$ corresponds to $6.3\hbox {e}^{-6}$ s. Thus, *Stk* is equal to $6.3\hbox {e}^{-5}$ and therefore for the conditions under which higher cell inertia is expected; cells will follow the fluid streamlines.

**Fig. 2:**
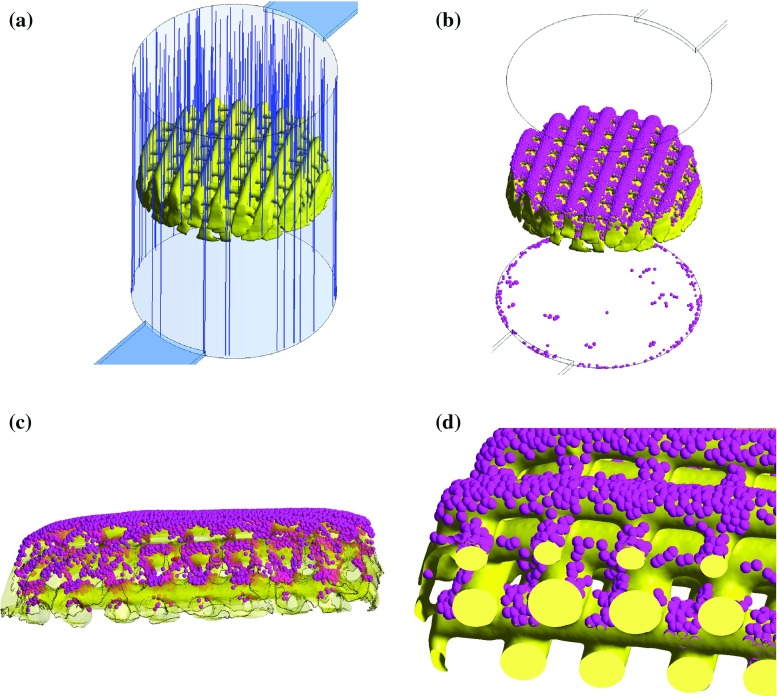
**a** Cell path from the injection surface at the top of the cylinder up to the first obstacle found. Cells travel with a constant velocity of 0.01 mm/s. **b** Cells attached to the scaffold or chamber after 2 h static seeding. The cells are represented with spheres ten times bigger than the real size of cells to improve visibility. **c** Side view of the scaffold with transparency applied in the fibres to visualize the internal distribution of cells from the top to the bottom layers. Most of the cells are found at the first layers as the last ones are covered by the ones on top. **d** Internal view of the scaffold fibres and cell distribution

**Fig. 3:**
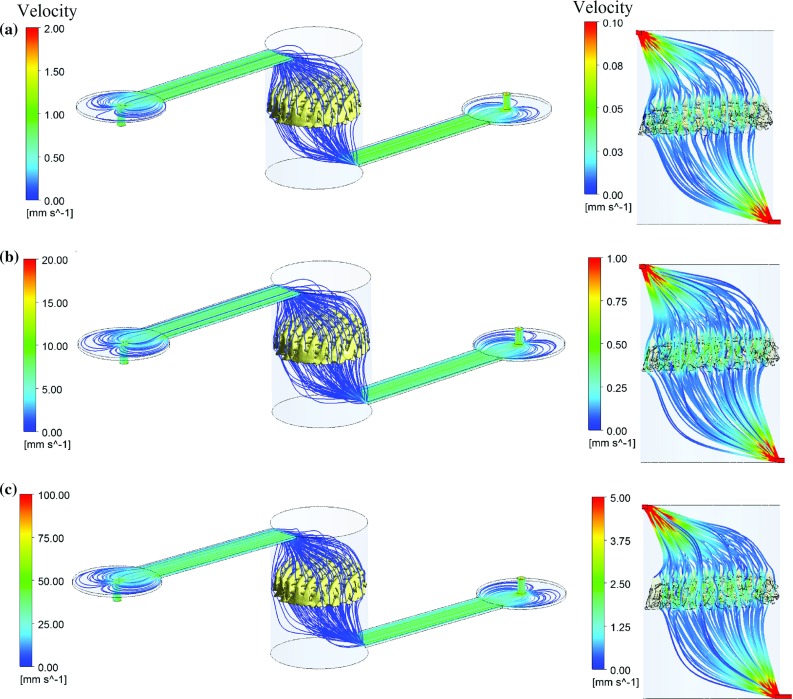
Fluid streamlines for the flow rates 12 (**a**), 120 (**b**) and 600 (**c**) $\upmu \hbox {l}$/min throughout the entire microfluidic system (left) and inside the cylinder and scaffold pores (right)

**Fig. 4:**
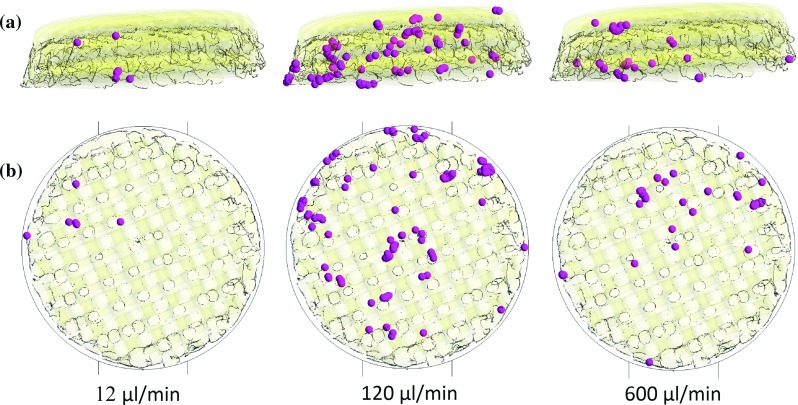
**a** Side view of the scaffold. Transparency is applied to the scaffold surface to visualize the cells attached inside the microstructure for the three flow rates applied after 2-h cell seeding. The cells are represented with spheres ten times bigger than the real size of cells to improve visibility. Irregular distribution of cells throughout scaffold depth is found for the three cases in the absence of gravity. **b** Top view of the scaffold. Irregular distribution of cells is found throughout scaffold diameter in all cases

**Fig. 5:**
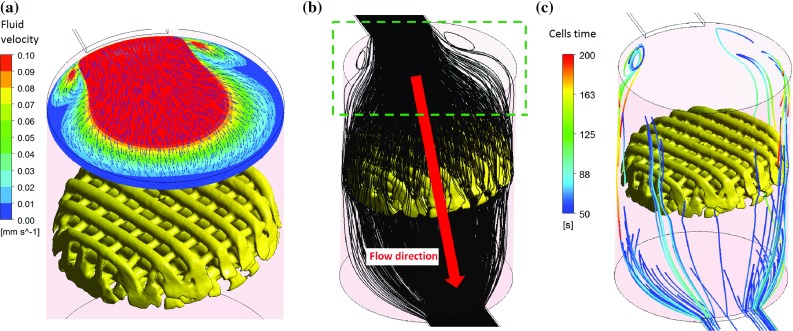
**a** Velocity profile above the scaffold showing the recirculation region formed for $600\,\upmu \hbox {l}$/min. **b** Cell path shows that cells are trapped inside these vortexes. **c** Cells do not cross the scaffold before changing the flow direction. Cells path are coloured from the time when the flow should be due to change direction up to 200 s (keeping the same flow direction). For $600\,\upmu \hbox {l}$/min, the flow should reverse at 50 s; however, cells are still recirculating in the vortexes

## Results

### Static seeding

In the static seeding, cells were injected from the top of the cylindrical chamber and they travelled down towards the scaffold due to gravity with a constant velocity of 0.01 mm/s. Cells advance following a straight path until they attach to the first obstacle they intercept on their way, either the scaffold substrate or the bottom of the chamber (see Fig. 2a). It is noteworthy to mention that cells are represented with spheres ten times bigger than the real size of cells in all figures to improve visibility. Cells attached to the scaffold fibres are found at the region that faces the surface of the microfluidic chamber where cells were injected. Thus, no cells are found at the opposite face of the fibres as seen in Fig. 2c. Despite the fact that 85% of cell seeding efficiency was found, there is no homogeneous distribution of cells throughout the scaffold microstructure. The majority of cells are attached on the top of the first, second and fifth layers as there are no obstacles along cell path from the injection point until these fibres. For the third and fourth layers, cells are only found at the sides of the fibres as these are aligned with the fibres on top, which cells encounter first. In the last layer of fibres, there are no cells as these fibres are completely covered by the ones above. Cells that do not intercept the scaffold substrate reach the bottom of the chamber through the gap between the scaffold and the chamber wall.

### Dynamic seeding

#### Fluid phase

12, 120, and $600\,\upmu \hbox {l}$/min were imposed at the inlet surface corresponding to 1, 10 and 50 mm/s of average velocity, respectively. The fluid velocity reduced two orders of magnitude from the inlet to the scaffold entrance since the fluid pass through an area hundred times larger than the inlet surface one. In all cases, the fluid streamlines pass homogeneously through the scaffold microstructure and the average velocity inside the scaffold pores is twice the average fluid velocity at the scaffold entrance (see Fig. 3).

The flow reverses every $500\,\upmu \hbox {l}$ of fluid dispensed; therefore, cells do not cross the scaffold the same number of times for different flow rates. $600\,\upmu \hbox {l}$/min is the highest flow rate which results in 144 cycles during the 2-h experiment as less time is required to dispense $500\,\upmu \hbox {l}$ than for 12 and $120\,\upmu \hbox {l}$/min flow rates with 3 and 28 cycles, respectively. As a consequence, cells are expected to cross the scaffold more times for higher flow rates increasing the probability for cells to intercept the scaffold substrate.

#### Cell phase in the absence of gravity

For the three flow rates applied, cells follow the fluid streamlines and their velocities are within the same values found for the fluid velocities. Under $12\,\upmu \hbox {l}$/min, where the flow reverses only twice and the secondary flow is weaker than in the other two cases, almost no cells attach on scaffold surface. Therefore, for $600\,\upmu \hbox {l}$/min, stronger secondary flow was expected and thereby higher cell deposition on scaffold substrate. However, scaffold cell deposition is higher for 120 than for $600\,\upmu \hbox {l}$/min flow rate as shown in Fig. 4.

The reason why for $600\,\upmu \hbox {l}$/min less cells deposit on the scaffold is the formation of two vortexes which are found inside the cylinder where the scaffold is located (see Fig. 5). These vortexes have a negative effect on cell seeding efficiency as cells are recirculating inside them when the flow is due to reverse. Thus, cells trapped in the vortexes will not cross the scaffold reducing the probability of cell deposition. Similar vortexes form underneath the scaffold when the flow reverses preventing again cells from crossing the scaffold. It is noteworthy that there are no preferable zones in the scaffold for cells to attach. Irregular distribution of cells is found for the three cases and in the case of $120\,\upmu \hbox {l}$/min where more cells are attached, cells are found throughout the scaffold depth.

#### Cell phase in the presence of gravity

The effect of gravity on cell motion becomes more significant for lower flow rates detaching cells from the flow stream. For the lowest flow rate $12\,\upmu \hbox {l}$/min, gravity has the strongest impact on cell motion where most of cells deposit in the microfluidic system as soon as the are injected (see “Appendix”, Fig. 10). The majority of cells deposit on the inlet channels just after the injection and before arriving to the cylindrical chamber where the scaffold is placed. Thus, only few cells reach the scaffold and the rest deposit on the bottom of the chamber. For $120\,\upmu \hbox {l}$/min, the transport of cells to the scaffold is enhanced and more cells deposit on it. However, most of the cells deposit in the channels and only few cells remain in suspension before reversing the flow for the first time. For $600\,\upmu \hbox {l}$/min, the effect of gravity on cell motion is negligible and cells mainly follow the fluid streamlines. Hence, cells are in suspension and cross the scaffold several times during the 2 h of seeding (see “Appendix”, Fig. 10). In spite of this, less cells deposit onto the scaffold in comparison with $120\,\upmu \hbox {l}$/min flow rate.

**Fig. 6:**
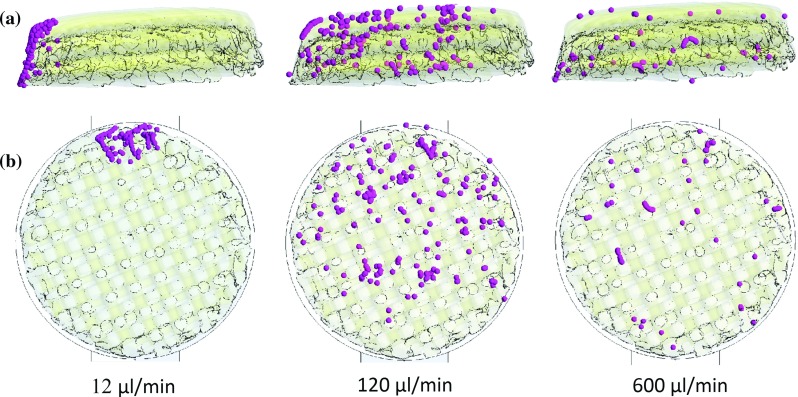
**a** Side view of the scaffold. Transparency is applied to the scaffold surface to visualize the cells attached inside the microstructure for the three flow rates applied after 2 h cell seeding. The cells are represented with spheres ten times bigger than the real size of cells to improve visibility. Irregular distribution of cells throughout scaffold depth is found for the three flow rate under the effect of gravity. **b** Top view of the scaffold. Irregular distribution of cells is found throughout scaffold diameter with more cells attached at the top corresponding to closest region to the injection surface

**Fig. 7:**
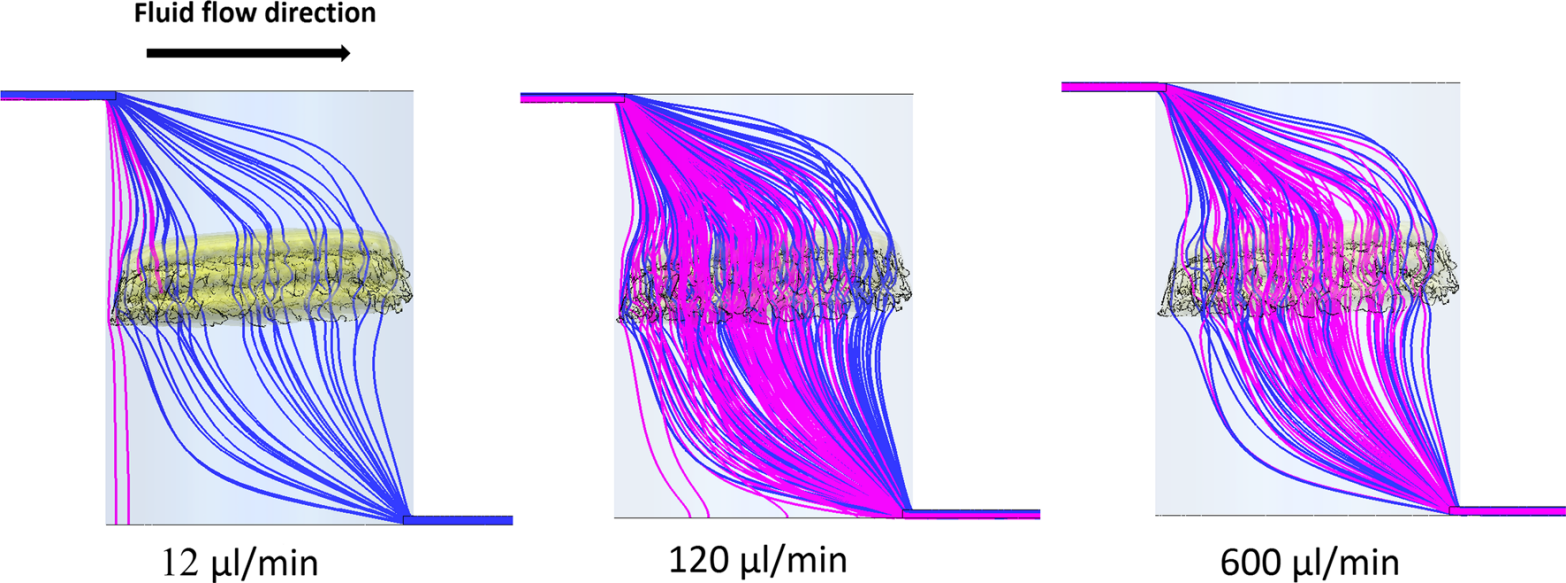
Cell path (pink) and fluid streamlines (blue) along the cylinder and scaffold pores (side view) for the three flow rates. Gravity effect on cell motion is stronger for lower flow rates especially for $12\,\upmu \hbox {l}$/min where cell path detaches from the fluid streamlines. For higher flow rates such as $600\,\upmu \hbox {l}$/min cells travel along the fluid streamlines as the effect of the gravity force is less strong

It is noteworthy that the distribution of cells attached to the scaffold is more homogeneous when seeding under $600\,\upmu \hbox {l}$/min than for the 120 and $12\,\upmu \hbox {l}$/min flow rates (see Fig. 6). This is due to the fact that for $600\,\upmu \hbox {l}$/min cells travel longer distances as they are less affected by gravity (see Fig. 7).

### In vitro versus in silico

**Fig. 8:**
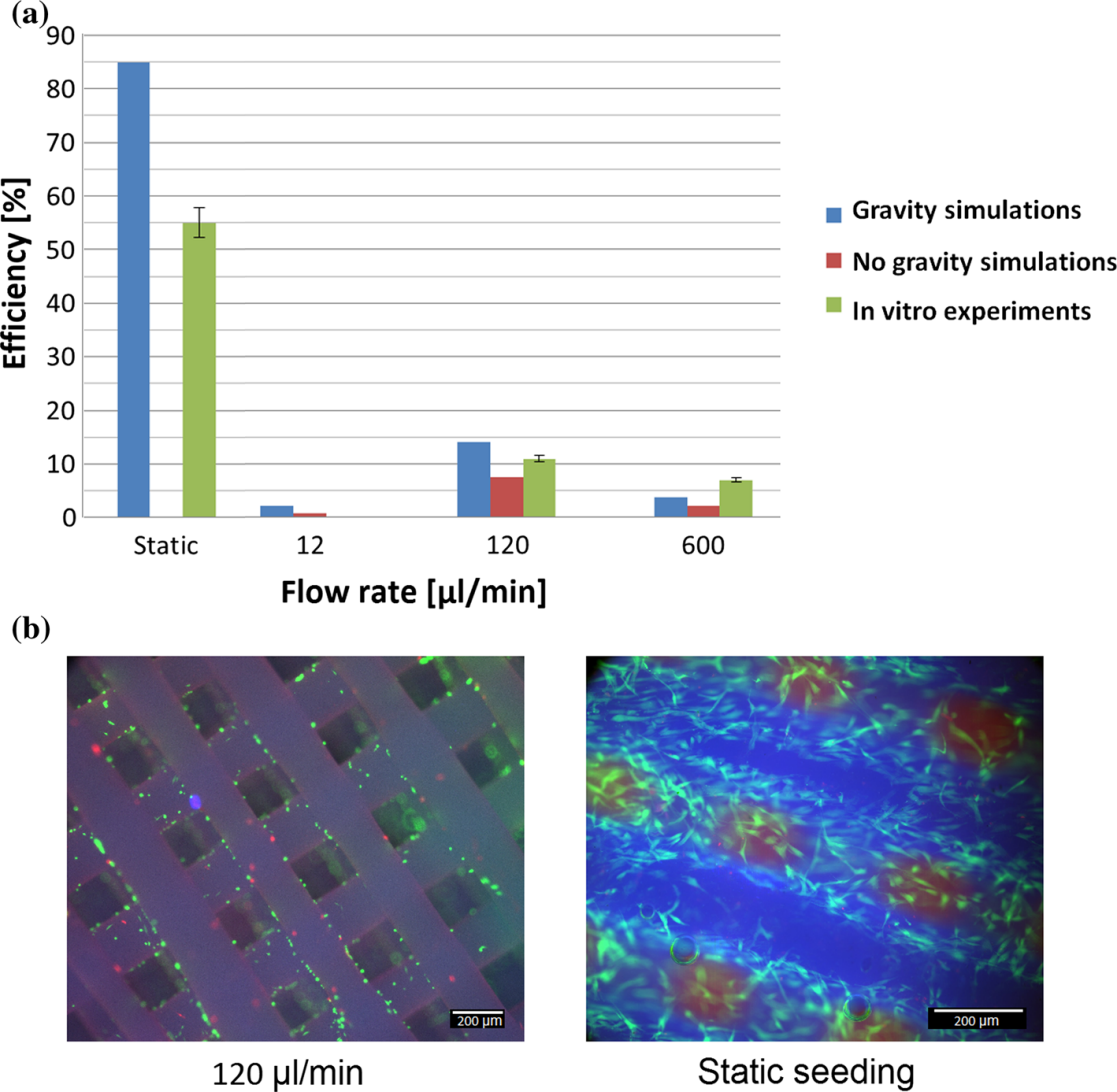
**a** Comparison of scaffold cell seeding efficiency between in vitro and in silico experiments at static, 12, 120 and $600\,\upmu \hbox {l}$/min flow rates. **b** In vitro cell seeding results for static seeding and dynamic seeding with $120\,\upmu \hbox {l}$/min flow rate. Final cell location and shape is observed within the scaffold for both cases

In vitro static cell seeding was performed in five scaffolds. The results show $55 \pm 5$% of efficiency whereas the simulations overestimates cell attachment with 85% efficiency (see results in Fig. 8a). In terms of cell distribution, both in vitro and in silico static results shows that cell deposition mainly occurs at the top of the scaffold (see Fig. 8b for in vitro and Fig. 2c for in silico).

In dynamic seeding, the highest efficiency found in the in vitro experiments 2 h is 11 ± 0.61% corresponding to $120\,\upmu \hbox {l}$/min flow rate. For $12\,\upmu \hbox {l}$/min no cells are found in the scaffold and for $600\,\upmu \hbox {l}$/min the efficiency results in $6.5 \pm 0.61$%. The same trend is found in the simulations whether gravity is accounted for or not; the highest efficiency is for $120\,\upmu \hbox {l}$/min and the lowest for $12\,\upmu \hbox {l}$/min (see Fig. 8a). After 24 h from in vitro dynamic seeding, there is less accumulation of cells at the top of the scaffold than in static seeding and a more uniform distribution of cells though the construct as also observed in the in silico results (see Fig. 8b for in vitro and Fig. 6 for in silico). Despite of this, their shape is round as observed in Fig. 8b suggesting low attachment.

## Discussion

A computational model was presented to understand how the fluid flow conditions during in vitro seeding affect cell transport and final scaffold seeding efficiency. In case of static seeding, cells sediment within the static fluid adhering to the first obstacle found. More cells are found at the fibres closer to the injection surface at the top of the chamber leading to a heterogeneous distribution of cells. In addition, the irregular distribution of fibres due to the inaccuracies in the manufacturing process (Campos Marin and Lacroix 2015; Brunelli et al. 2017a) also contributes to the heterogeneous spatial distribution of cells attached to the scaffold. It is noteworthy that cells were only found at the scaffold side that faces the injection region at the top of the chamber. A heterogeneous distribution within the scaffold should be avoided since this could lead to poor mineralization affecting viability and mechanical properties. If the chamber was rotated, so the bottom face becomes the top and thereby the injection face, an additional injection at this side could improve the cell spatial distribution. However, in case of larger scaffolds with more clinically relevant dimensions, the transport of cells to the interior of the scaffold will be challenging and other solutions should be explored.

For dynamic seeding, it was found that cells follow the fluid streamlines if gravity is not considered. This was also observed experimentally in a previous study of Campos Marin et al. (2017) under higher flow rates which avoids the effect of gravity. This is due to the low ratio between cell time response and the characteristic time of the fluid. Therefore, if cells cannot detach from the flow, the fluid should flow towards the scaffold substrate for cells to deposit on it. For higher flow rates, secondary flow becomes more significant so more cells can be dragged towards the scaffold and impact onto the substrate. This can especially occur when there are sudden changes in the geometry along the fluid path. Thus, higher efficiencies were expected for higher flow rates. However, above a certain flow rate, vortexes can form trapping cells and preventing them from passing through the scaffold and depositing on it.

When gravity is considered, cells deviate from the fluid streamlines for lower flow rates. Thus, gravity is one of the mechanisms that can bring cells to intercept with the scaffold. The lower the fluid velocities the higher is the effect of gravity, however, too low fluid velocities can result in poor transport of cells towards the scaffold. Therefore, a trade-off between fluid velocities and force due to gravity needs to be found to increase cell seeding efficiency.

It is important to mention that each flow rate results in different number of cycles during the 2 h of seeding as they require different amount of time to dispense $500\,\upmu \hbox {l}$. Lower flow rates result in less number of cycles, thereby reducing the number of times that cells cross the scaffold. This could be improved by increasing the number of cycles although that would require longer experiments. In fact, the long exposure of cells to shear stress can be detrimental for cell viability or induce cell detachment (Deligianni et al. 2000).

A good agreement between in silico and in vitro cell seeding efficiencies was found. However, five scaffolds were seeded in vitro for each fluid flow conditions whereas only one scaffold was studied computationally for all cases. Repeated simulations should be performed on $\upmu \hbox {CT}$-based scaffolds from different samples in order to gain statistical significance.

Both methods show that static seeding is the most adequate option for such scaffold and microfluidic chamber. However, static seeding efficiency in the simulations was 35% higher than in the experiments. That can be due to the limitation of the model to simulate realistic cell adhesion events or formation of cell clusters. Another reason could be the fact that cells were injected with a regular distribution over the injection surface in the simulations, whereas in the in vitro experiments a drop of media with cells is placed on top of the scaffold where the initial cell distribution is unknown. A non-regular initial distribution could promote the formation of clusters and a more irregular distribution of cells after seeding thereby affecting cell viability. Also, in spite of static seeded scaffolds showing high density of spread cells on the top surface, the red signal coming from the interior of the scaffold seen in Fig. 8b suggests enhanced cells death in the internal volume probably due to lack of gases and nutrients exchange.

In dynamic seeding, the same trend was found between experiments and simulations in terms of flow rate and seeding efficiency, both with and without gravity. When the simulations account for gravity, the force due to gravity helps cells to detach from the flow stream and impact on the scaffold, if the adequate flow rate is applied and the good transport properties are met. When gravity is neglected, the flow rate determines whether secondary flow carries cells towards the walls. In summary, gravity and secondary flow are key factors for cell deposition. However, they have been explored separately. As a consequence, the real contribution of each of them on cell deposition during in vitro experiments is still unknown. Moreover, different cell density values will alter the influence of gravity on cell transport leading to different results. Nonetheless, the inclusion of the cell density in the computational model results in a more cell-type specific in silico tool.

In the computational model, cells adhere to the boundary as soon as they intercept it. Hence, this model can only report the maximum cell seeding efficiency. In vitro experiments are more complex scenarios where cell adhesion is not always guaranteed and this could explain some of the differences reported between in vitro and in silico seeding efficiencies. For instance, cells that contact the scaffold substrate may not adhere to it due to the biocompatibility with scaffold material (Bačáková et al. 2004), unsuitable mechanical properties of the substrate (Kawano et al. 2013) or surface roughness (Viswanathan et al. 2015). Furthermore, cells that are already adhered can detach from the scaffold if they are exposed to high fluid-shear stresses provoking the rupture of cell focal adhesions (Tang et al. 2012; McCoy et al. 2012). Moreover, the model does not capture cell viability of adhered cells which needs to be ensured so the next steps for the formation of new tissue such as proliferation and differentiation can occur. It is noteworthy that scaffold coating is generally used during in vitro cell seeding to promote cell adhesion (Lan et al. 2009) and other forces may also play a role near the scaffold surface for cell attachment. However, these forces would have to overcome forces due to fluid drag in order to deviate cells from the fluid streamlines. Spencer et al. (2013) included cell adhesion events in a computational model for cell seeding by modelling receptor–ligand dynamics in the first monolayer of cell adhesion. However, cells were not computed individually and as they were represented by a suspension concentration.

Cells were set to reflect at the inlet and outlet boundaries preventing them from escaping from the fluid domain. Nevertheless, in the in vitro experimental scenario, cells travel out of the chamber towards the syringe and may not be back inside the microfluidic chamber and therefore cross the scaffold pores again. Consequently, the model calculates cell seeding efficiency out of all cells that were initially injected so it can overestimate seeding efficiency results.

The fact that cell-to-cell interactions were not modelled could also explain the differences found between in silico and in vitro efficiencies. They were not included due to the low fraction of volume that the cell phase represented in the fluid phase. This assumption can be critical when cells are driven to the same scaffold region. In the computational model, cells can overlap and attach at the same location. However, in the experiments, cells can form clusters which can alter cell response during seeding and the first stages of tissue formation. In addition, clusters of cells at the scaffold surface become a new physical boundary with higher surface area for cells to attach. Moreover, this new boundary can alter the local fluid dynamics. Consequently, the formation of clusters can have an important impact on cell seeding and this could be further investigated with moving boundary methods such as the level set method (Guyot et al. 2015).

**Fig. 9:**
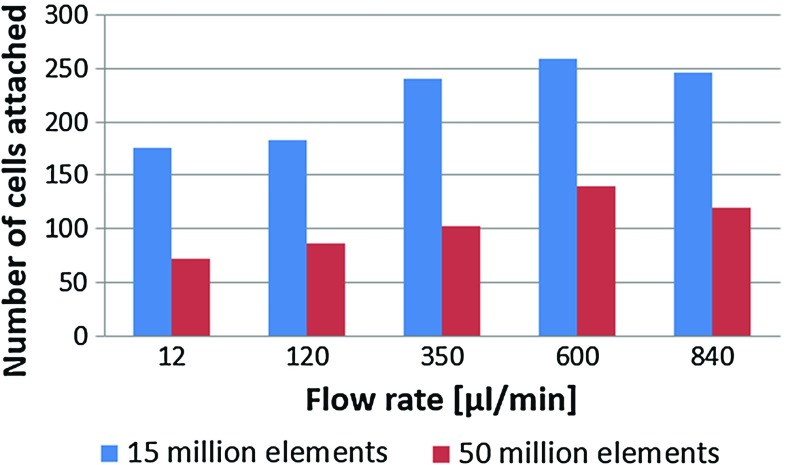
The fluid flow simulations converge for a mesh with around 15 million tetrahedral elements. However, the influence of the mesh size on the discrete phase calculations should be explored. A comparison of the discrete phase predictions between meshes with 15 and 50 million elements was carried out. A simple discrete phase model case was applied to compare the performance of both meshes; a single injection was set at the inlet and 15 cycles were applied to transport the cells towards the scaffold and the outlet and then back consecutively. 12, 120, 350, 600, $840\,\upmu \hbox {l/min}$ flow rates were applied. In all the cases the model with 50 million elements mesh reports lower cell deposition. As a result, the 15 million elements mesh converges for the fluid phase but not for the discrete phase

**Fig. 10:**
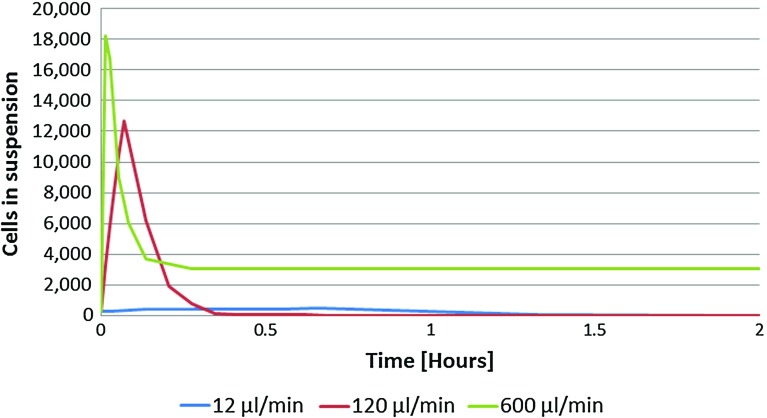
Number of cells in suspension in the fluid during the 2 h of cell seeding experiment for the three flow rates investigated when gravity is considered

It is important to note that the density of the computational mesh can affect the deposition of the discrete phase on the wall boundaries. Thus, different mesh convergence studies should be carried out for the discrete and the carrier phases as shown suggested by Frank-Ito et al. (2015). They introduced a two-stage mesh refinement approach to simulate accurately the deposition of particles on human sinonasal cavities for post-surgery nebulized drug delivery applications. The transport of particles in airflow was modelled using the discrete phase model of Fluent Ansys, as in the present study, in different unstructured tetrahedral meshes with varying density. They found that the discrete phase required higher mesh density to converge than the air/carrier phase as also shown in the mesh convergence study carried out in this study (see “Appendix”, Fig. 9). The discrete phase does not converge for the mesh employed in the simulations and cell deposition was overestimated. Unfortunately, the computational cost required to solve the full model with denser meshes becomes unaffordable. This affects the predictions for cell seeding efficiency although the same efficiency trends found in the in vitro experiments were reported.

Another aspect to be further explored in the computational model is the advancement of cells when the fluid flow reverses direction. While the flow direction is reversed, fluid velocities are decreased and then increased again to move in opposite direction. As cells respond rapidly to changes in the fluid flow, cell velocity will also be reduced and later increased following the fluid flow. If cell velocity is reduced during this transition, gravity force can overcome fluid drag forces and induce cell deposition. However, in the present model, the fluid phase at each time step was resolved in the opposite direction and completely developed before cells were advanced in the fluid domain. As a result, the impact of reversing the fluid flow until reaching a steady state on cell transport was not captured.

Initially, the dynamic approach was expected to deliver higher efficiency than the static seeding due to the excellent mass transport properties of perfusion systems. In the literature, different studies have shown the benefit of seeding scaffolds using this approach (Li et al. 2001; Wendt et al. 2003; Zhao and Ma 2005; Alvarez-Barreto et al. 2007; Zhao et al. 2007). Nonetheless, these scaffolds have an irregular porous microstructure whereas in this study, an additive manufacturing scaffold with a regular design was investigated. More tortuous channels can enhance secondary flow and cell deposition on scaffold substrate. Also, well-defined open porous structures will lead to regular flow profiles dragging cells along the centre of the pores without intercepting the scaffold surface. Thus, the number of cells that intercept in additive manufacturing scaffolds during perfusion can be significantly lower than for static seeding as seen herein and by Bartnikowski et al. (2014).

The silico tool holds the potential to help researchers to stop seeing perfusion seeding systems as black boxes and optimize them to enhance their efficiency. In fact, this study shows that perfusion systems enhance cells transport to the scaffold but do not guarantee the interception of cells with scaffold substrate. Therefore, more efforts should be made to promote cell-scaffold interception so cell adhesion may occur. For instance, a rotating perfusion system chamber such as the one presented by Melchels et al. (2010) or Haykal et al. (2014) could increase cell impaction. Another approach would be the one presented by Papadimitropoulos et al. (2013) where a collagen network was cross-linked within the scaffold pores to enhance cell entrapment and cell differentiation as shown by Brunelli et al. (2017b).

## Conclusions

A computational model able to predict scaffold cell seeding over time and under different fluid flow conditions was presented. This model was used to explore an in vitro system aimed to seed cells into a 3D porous scaffold following static and dynamic approaches. On the one hand, static seeding where cells are driven by the force due to gravity showed the best efficiency. Nonetheless, an irregular distribution of cells inside the scaffold was found due to their difficulty to reach deeper regions, which would be more significant in case of larger scaffolds. On the other hand, dynamic seeding by perfusion can overcome the poor transport properties of static seeding. However, in both experimental and computational studies, too low efficiencies were found in the dynamic seeding due to the fact that cells follow the fluid streamlines closely without intercepting the scaffold substrate. The present study suggests that only secondary flows or the effect of gravity can drive cells towards the scaffold under fluid flow. This study shows that the selection of the flow rate is an important factor that determines the actual contribution of these driving mechanisms on cell deposition. The presented in silico model is a powerful tool that can reduce the number of expensive trials and cell waste by providing a quantitative understanding of bioreactor/scaffold and cell interactions. This study suggests that perfusion systems are not the most adequate seeding technique for scaffolds with open regular porous geometries so new approaches should be pursued to promote cell-scaffold interception and thereby cell adhesion.

## Appendix

See Figs. 9 and 10.
